# DyAMNet: dynamic adversarial and contrastive network for EEG biometrics

**DOI:** 10.3389/fnins.2026.1815191

**Published:** 2026-06-26

**Authors:** Ting Li, MengFan Li, Ran Sun, JingJing Feng

**Affiliations:** 1 School of Computational Science and Computer Science, Xi’an Polytechnic University, Xi’an, China; 2 Shaanxi Key Laboratory of Clothing Intelligence, School of Computer Science, Xi’an Polytechnic University, Xi’an, China

**Keywords:** biometric recognition, contrastive learning, dynamic adversarial learning, electroencephalogram (EEG), identity authentication

## Abstract

**Introduction:**

Electroencephalogram (EEG)-based biometric recognition for brain–computer interfaces faces challenges from domain shifts, temporal nonstationarity, and limited scalability.

**Methods:**

To address these issues, we present DyAMNet, a framework that combines EEG microstate analysis with a hybrid attention mechanism. DyAMNet employs dynamic loss balancing to improve generalization and constructs a domain-invariant feature space that supports user expansion without catastrophic forgetting. We evaluated the model on three benchmark datasets (DEAP, THU-EP, and SEED).

**Results:**

The framework attains 87.2% accuracy in cross-dataset recognition and retains 84.0% accuracy when incrementally scaling to 60 users. The system also tolerates physiological artifacts and intersession signal drift, outperforming state-of-the-art models.

**Discussion:**

These findings show that dynamic adversarial training coupled with contrastive feature learning reduces brain-signal variability and preserves scalability. The work establishes a robust basis for feasible identity authentication and supports deploying brain–computer interfaces in clinical and everyday settings. The code is available at: https://github.com/cangtianhaoxue/DyAMNet.git.

## 1 Introduction

Brain-computer interface (BCI) systems require reliable user authentication to ensure secure access and protect patient safety. EEG-based biometric recognition has emerged as a promising solution due to its inherent liveness detection (Eraslan et al., 2025) and resistance to forgery. However, transitioning such systems from laboratory settings to real-world clinical or everyday environments remains challenging. Specifically, three interconnected issues must be addressed: (1) domain shifts caused by biological artifacts (e.g., EOG, EMG) and signal drift across sessions (Eraslan et al., 2023); (2) temporal non-stationarity of EEG signals, which makes identity-relevant features highly variable over time; and (3) scalability constraints, as practical systems must support dynamic enrollment of new users without retraining or catastrophic forgetting (Finn et al., 2015; Azuma et al., 2023). Addressing these challenges collectively is essential for deploying robust and scalable EEG-based authentication in real-world BCI applications.

EEG-based authentication encounters three interconnected challenges stemming from the nature of neural signals and clinical practices. First, domain shifts in clinical settings pose a problem: EEG recordings are influenced by biological artifacts such as EOG and EMG, and signal drift can occur due to neural plasticity, variations in patient states, and changes in electrode impedance across different sessions (Tian J. et al., 2025; Cai et al., 2023). Second, the non-stationary temporal characteristics of EEG mean that identity-discriminative features are embedded in time-series data that exhibit variability. Third, practical systems must be capable of dynamically adding new users without necessitating retraining or experiencing catastrophic forgetting, which is crucial for scalable clinical deployment.

Existing studies typically tackle these challenges either individually or through pairwise combinations. In addressing domain shift, adversarial learning has evolved from domain classification to techniques like progressive decision boundary sharpening (Shi et al., 2025; Ding et al., 2023), often integrating pseudo-labeling for unsupervised adaptation (Tian J. et al., 2025; Del Pozo-Banos et al., 2014). For modeling EEG temporal dynamics, contrastive learning is employed to acquire discriminative and structured features (Ozturk et al., 2025; Tian Q. et al., 2025). To combat catastrophic forgetting in incremental learning, strategies include mitigating data imbalance (Liu et al., 2024) and employing neuroscience-inspired online learning mechanisms (Lin et al., 2025). The integration of domain adaptation and continual learning is used to manage temporally evolving data distributions (Yao et al., 2025), with one module removing session-specific confounders and another consolidating new knowledge for BCI systems.

Current studies reveal a research gap where domain generalization, temporal modeling, and incremental expansion are treated as separate issues. However, in real biomedical applications, these challenges coexist and interact. Systems must authenticate users with limited initial calibration data using non-stationary signals while seamlessly integrating new patients. Isolated solutions fall short of meeting these demands. Therefore, a unified learning framework is essential to address three core challenges: (1) achieving cross-domain generalization with limited source data, (2) extracting temporally stable features from non-stationary sequences, and (3) constructing a structured feature space for efficient and stable expansion to new classes.

This paper introduces the EEG-based identity recognition learning model using Dynamic Adversarial and Contrastive Learning (DyAMNet), specifically tailored for biomedical engineering applications. Although the datasets involve emotion evocation, real-world BCI authentication rarely occurs in a pure resting state. Therefore, robustness to emotional fluctuations is a practical requirement. Our evaluation focuses on emotion-tolerant identity recognition and does not claim complete stimulus independence, which is left for future work. This study’s primary contribution is the integration of algorithm design with the practical needs of biomedical applications. DyAMNet combines dynamic adversarial alignment, hybrid attention temporal modeling, and contrastive feature learning to address EEG-based identity authentication in biomedical BCI contexts. By optimizing adversarial loss and identity contrast loss, the model learns a domain-invariant feature space grounded in neural dynamics, which sharpens feature structure both within and between classes. The approach enables progressive expansion of the user base without relying on replay mechanisms, offering a practical technical path toward clinically scalable BCI authentication.

The paper is structured as follows: Section II reviews related work on domain adaptation in biomedical signal processing, temporal modeling of non-stationary EEG, and scalable biometric system design. Section III details the DyAMNet framework, focusing on EEG microstate feature extraction grounded in neurophysiological principles and the core model architecture. Section IV presents experimental results, analyzing the framework’s performance and internal mechanisms. Section V concludes the paper and suggests future research directions for clinical translation.

## 2 Materials and methods

### 2.1 Datasets and preprocessing

The DEAP dataset (Koelstra et al., 2012) is a multimodal database for emotion analysis using EEG, peripheral physiological signals, and facial video recordings, with 1,280 valid trials. The THU-EP dataset (Hu et al., 2022) performs fine-grained emotion recognition from EEG, with stimulus materials including target emotions like anger and joy, and has 2,240 valid trials. The SEED dataset (Zheng and Lu, 2015) features an ecologically valid setup using movie clips to evoke emotions, collecting EEG and eye movement recordings for studying emotional stability across sessions, with 675 valid trials. Some details of the three data sets are shown in Table 1.

**Table 1:** Details of the three data sets.

Data set	DEAP	THU-EP	SEED
Sampling frequency (Hz)	128	250	200
Single trial duration (s)	60	30	240
Trial count	1,280	2,240	675

### 2.2 EEG microstate feature extraction

In this study, three EEG datasets were standardized and preprocessed, and cross-subject microstate sequences were extracted. The datasets—DEAP (32 channels), SEED (62 channels), and THU-EP (32 channels)—were combined while preserving their original channel configurations. After band-pass filtering, the EEG signals were decomposed into three frequency bands: *δ* (0.5–3 Hz), *θ* (4–7 Hz), and *α*(8–13 Hz). This selection is based on our target application—identity authentication under natural, non-resting conditions. Lower frequency bands are less susceptible to real-time muscle/ocular artifacts and maintain stable individual specificity across sessions, while *β*/*γ* oscillations are strongly modulated by transient emotional and motor activity, introducing adverse domain shifts. All data were then converted into a uniform two-dimensional matrix format of time points × number of channels.

**Figure 1:**
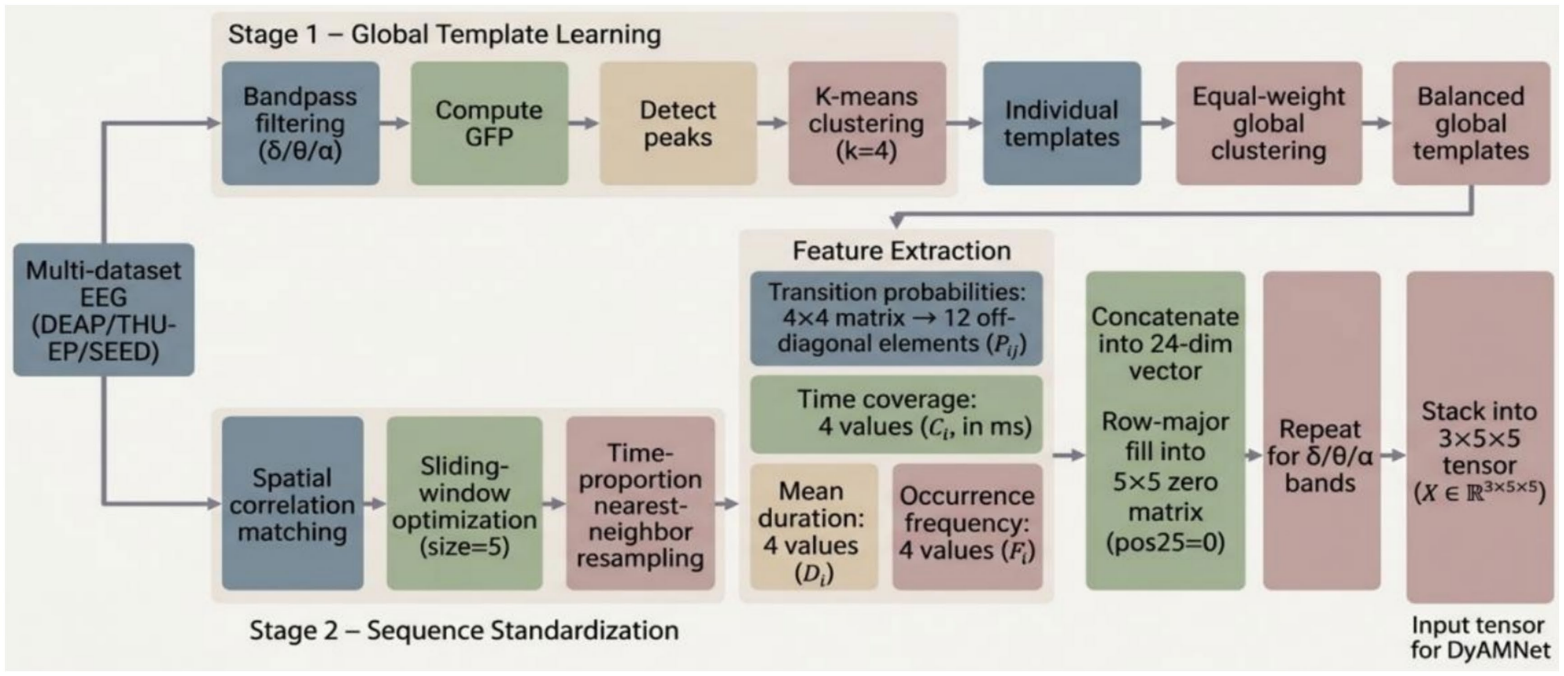
Microstate sequence computation flow.

We adopt a two-stage progressive computational framework to address across-dataset heterogeneity (showed in Figure 1). In the first stage, global template learning proceeds by calculating the global field power (GFP) for each subject and identifying peak points. K-means clustering (*k* = 4) then generates individual microstate templates. To produce balanced global templates that are independent across datasets, we apply an equal-weight strategy that randomly selects up to 100 templates from each dataset for global clustering. In the second stage, sequence standardization assigns each time point to the most relevant microstate category via spatial correlation. A sliding-window optimization algorithm (window size = 5) suppresses abnormal state transitions. Finally, we normalize sequence lengths using a time-proportion nearest-neighbor resampling method (DEAP: 8,064 points, THU-EP: 7,500 points, SEED retains the original length).

We systematically extract four classes of spatiotemporal features from the standardized microstate sequences to form a 24-dimensional feature vector. The first class comprises state-transition probabilities. We build a 4 × 4 state-transition count matrix:


$$C_{\mathrm{ij}}=\sum_{t=1}^{T-1}I(s_{t}=i,s_{t+1}=j)$$ (1)

and compute the corresponding transition-probability matrix


$$P_{\mathrm{ij}}=\frac{C_{\mathrm{ij}}}{\sum_{k=1}^{4}C_{\mathrm{ik}}}.$$ (2)

The 12 off-diagonal elements $\left\{P_{\mathrm{ij}}|i\neq j,i,j\in \{0,1,2,3\}\right\}$ are taken as features.

The second class is time-coverage. For each microstate class i (yielding 4 features), compute the proportion of total time occupied:


$$C_{i}=\frac{\sum_{t=1}^{T}I(s_{t}=i)}{T}\times 100\%.$$ (3)

The third class is mean duration. We detect consecutive segments of each state and convert their lengths to physical time (milliseconds):


$$D_{i}=\frac{{\bar{L}}_{i}}{f_{s}}\times 1,000,$$ (4)

where ${\bar{L}}_{i}$ is the average segment length in samples and $f_{s}$ is the sampling rate.

The fourth class is occurrence frequency. We compute the rate of occurrences per unit time:


$$F_{i}=\frac{N_{i}}{T/f_{s}},$$ (5)

where $N_{i}$ represents the number of occurrences of the state i . This yields another 4 features.

The four groups of features are concatenated in sequence to form a 24-dimensional feature vector:


$$f=[P_{\mathrm{off}-\text{diagonal}},C,D,F],\text{where}P_{\mathrm{off}-\text{diagonal}}\in R^{12},C,D,F\in R^{4}$$ (6)

To match the input format required by the convolutional neural network, the 24-dimensional feature vector is reshaped into a 5 × 5 matrix. A 5 × 5 zero matrix $M\in R^{5\times 5}$ is initialized, with $M_{5,5}=0$ used as the padding placeholder. Following row-major order, the 24 feature values are placed sequentially into the first 24 entries:


$$M_{i,j}=f_{k},\text{where}k=5(i-1)+j,i=1,\ldots ,5,j=1,\ldots ,5,k=1,\ldots ,24$$ (7)

Design a matrix layout that preserves clear physical meaning. Fill the first 12 positions with state-transition probability features. Reserve positions 13–16 for average-duration features ( $D_{A},D_{B},D_{C},D_{D}$ ), positions 17–20 for appearance-frequency features ( $O_{A},O_{B},O_{C},O_{D}$ ), and positions 21–24 for time-coverage features ( $C_{A},C_{B},C_{C},C_{D}$ ). Leave position 25 as a zero-filled placeholder. This arrangement produces a structured spatial representation while preserving the physical interpretation of each feature. These features are computed using the formulas defined in Equations (1–7).

For each trial, three frequency bands are processed independently and stacked into a three-dimensional input tensor. Five-by-five feature matrices for the δ, θ, and α bands are generated and stacked as $X\in R^{3\times 5\times 5}$ so that the first dimension represents the frequency-band channels. We constructed a standard dataset of 4,195 samples from 127 subjects. Each sample contains three components: a feature tensor (a 3 × 5 × 5 microstate feature matrix), a unified subject ID label, and a source dataset identifier.

### 2.3 DyAMNet model

**Figure 2:**
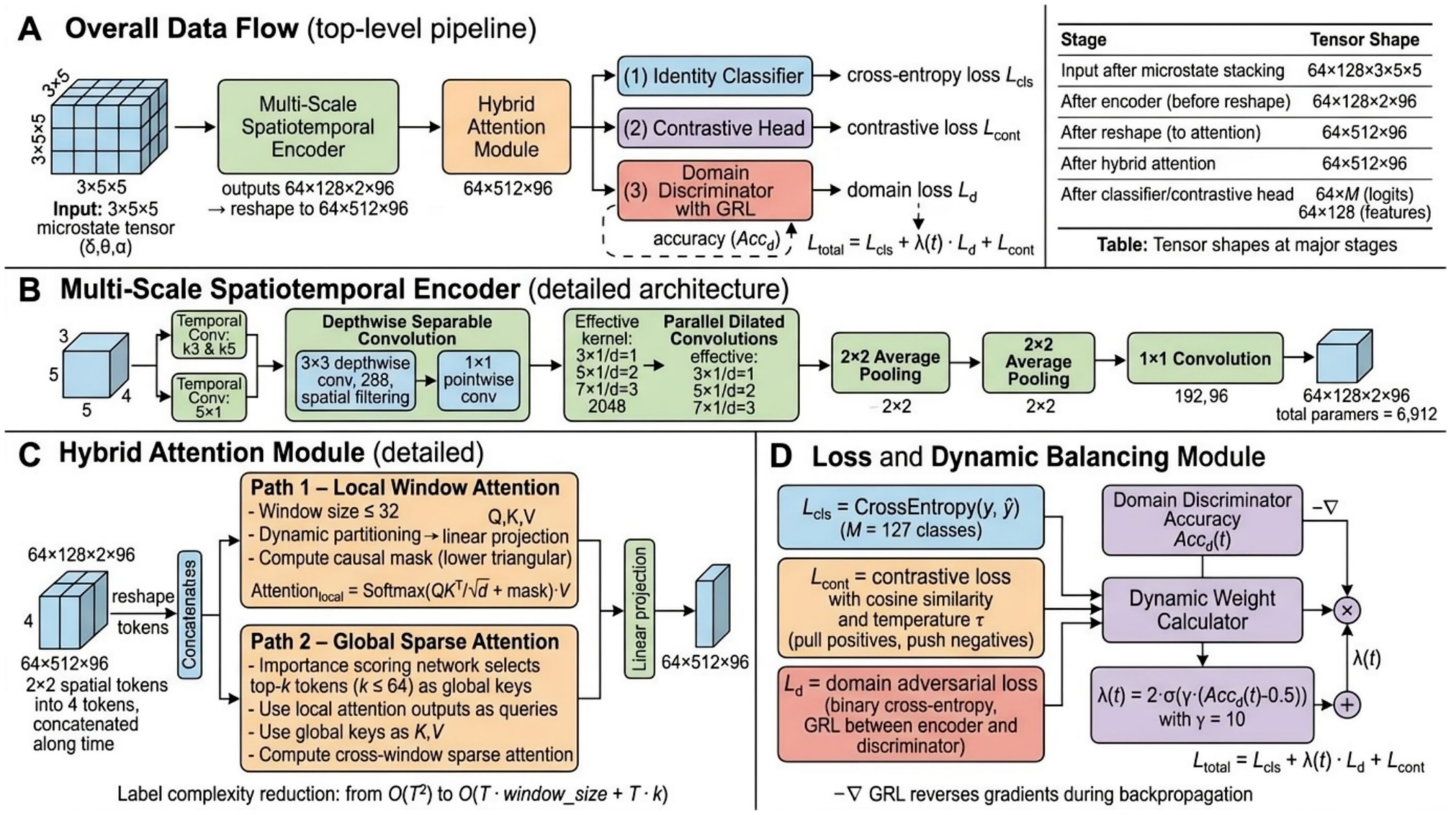
The DyAMNet for EEG-based identity recognition. **(A)** Multi-scale spatiotemporal encoder extracts temporal and spatial features from microstate inputs. **(B)** Hybrid attention module models long-range dependencies via local and global attention. **(C)** Domain adversarial learning dynamically balances domain invariance and identity discrimination. **(D)** Contrastive consistency loss enforces intra-class compactness and inter-class separation.

The DyAMNet is a deep learning model developed to tackle cross-domain individual identification tasks utilizing electroencephalography (EEG) data (as illustrated in Figure 2). The main contribution of this study lies in integrating multiple advanced techniques-dynamic adversarial alignment, hybrid attention, and contrastive learning-into a unified framework for EEG-based biometric recognition. To this end, we construct a unified framework that amalgamates multi-scale spatiotemporal encoders, hybrid attention modules, and contrastive bottlenecks during the encoding and decoding stages, alongside domain-specific adversarial training.

#### 2.3.1 Input representation

The model input is a 4D tensor built from the electroencephalogram (EEG) microstate feature matrix to represent individual physiological patterns. The batch size is 64, and each sample contains 128 consecutive time steps (representing 128 independent trial observations of the same subject). Each time step is a 5 × 5 matrix (derived from 24-dimensional microstate features plus one zero-padding bit) and maps to the *δ*, *θ*, and *α* frequency bands across three channels. This arrangement allows the model to extract robust, discriminative subject-specific features from multiple frequency bands and to capture dynamic neurophysiological changes during task execution.

#### 2.3.2 Multi-scale spatiotemporal encoder

The model input is a 4D tensor (batch size × 128 time steps × 5 × 5 matrix × 3 frequency bands). At each time step, the 5 × 5 matrix contains 24-dimensional microstate features from a single trial plus 1 zero-padding bit. The encoder uses a spatiotemporal decomposition architecture. In the temporal domain, parallel one-dimensional convolutions employing kernel sizes of 3 × 1 and 5 × 1 are applied to capture multi-scale temporal features. For spatial encoding, a two-stage depthwise separable convolution structure is employed: first, a 3 × 3 depthwise convolution with 288 groups performs spatial filtering, followed by a 1 × 1 pointwise convolution comprising 2048 parameters. This design facilitates the extraction of spatial features from three distinct frequency bands, followed by their integration through feature fusion. Multi-scale expansion is implemented by parallel dilated convolutions with effective kernels 3 × 1/*d* = 1, 5 × 1/*d* = 2, and 7 × 1/*d* = 3. The output is downsampled to 2 × 2 using 2 × 2 average pooling, and channel dimensionality is reduced from 192 to 96 via a 1 × 1 convolution. This design naturally removes the influence of the zero-padding through the pooling operation after feature reshaping, and the model contains 6,912 parameters in total.

#### 2.3.3 Hybrid attention module

First, reconstruct the input. The encoder output, a four-dimensional feature tensor $X\in R^{64\times 128\times 2\times 96},$ is reshaped into a three-dimensional tensor. The two spatial dimensions (2 × 2 = 4 spatial tokens) are collapsed into a single token and then concatenated along the temporal axis to yield 512 time steps (128 original time blocks × 4 spatial tokens), producing the reconstructed tensor $X^{\prime }\in R^{64\times 512\times 96}.$ Therefore, the temporal axis in the hybrid attention module operates on the trial-level microstate summary sequence (128 consecutive trials per subject), not on intra-trial dynamics.

Next, perform mixed-attention computations on the reconstructed feature tensor $X^{\prime }$ . Begin with local window attention: adapt the window size dynamically so that at least one window is formed, partition the sequence into multiple windows (each window size ≤ 32), and compute causal self-attention within each window. Generate queries, keys, and values by linear projection, and compute attention weights ${\text{Attention}}_{\text{local}}(Q,,K,,V)=\text{Softmax}({\mathrm{QK}}^{T}/\sqrt{d})V\;$ using the scaled dot-product mechanism with a dynamically constructed lower-triangular causal mask matrix M .

Compute global sparse attention in parallel. Assess each token’s importance with the importance-scoring network, then select the top k tokens with the highest scores as global key tokens (*k* ≤ 64). Use the local-attention outputs as queries and the global key tokens as keys and values to compute sparse attention across windows. Finally, merge the two attention outputs and apply a linear projection to produce the final result. This design reduces the computational complexity from O(T2) to $O(T\cdot \text{windo}w_{\_}\text{size}+T\cdot k)$ , enabling efficient long-sequence modeling while preserving causality.

#### 2.3.4 Identity classification loss

To ensure the discriminative power of subject identity, we adopted standard cross-entropy loss on M subject categories. *M* = 127 in our experiments, corresponding to the total number of subjects across datasets.

Classification Loss as shown in Equation (8):


$$L_{\mathrm{cls}}=-\frac{1}{N_{b}}\sum_{i=1}^{N_{b}}\sum_{c=1}^{M}y_{i}^{c}\log({\hat{y}}_{i}^{c})$$ (8)

N_b_: Batch size 64.

M: Number of categories (127).

$y_{i}^{c}$ : true label (one-hot encoded) for sample i.

${\hat{y}}_{i}^{c}$ : model’s predicted categorical probability distribution.

#### 2.3.5 Domain adversarial loss

To encourage learning of domain-invariant features, we introduce a domain discriminator $D_{d}$ that predicts whether a feature originates from the source or the target domain. A gradient reversal layer (GRL) is placed between the encoder and the discriminator; the GRL acts as an identity mapping in the forward pass but negates gradients during backpropagation. This design induces an adversarial game between the encoder and the discriminator. The domain adversarial loss is defined as shown in Equation (9):


$$L_{d}=-\frac{1}{N}\sum_{i=1}^{N}[d_{i}\log({\hat{d}}_{i})+(1-d_{i})\log(1-{\hat{d}}_{i})]$$ (9)

Here, $d_{i}\in \{0,1\}$ : domain label (0: source domain, 1: target domain). ${\hat{d}}_{i}$ : domain discriminator prediction probability.

#### 2.3.6 Contrastive consistency loss

To preserve intra-class compactness and inter-class separation in the feature space, particularly under cross-domain conditions, this study adopts a contrastive learning objective. For each anchor sample i, the model pulls its feature $z_{i}=E(x_{i})$ closer to features of the same subject from any domain [positive sample set P(i)] and pushes it away from features of all other subjects [negative sample set N(i)] as defined in Equation (10):


$$L_{\text{cont}}=-\frac{1}{|P|}\sum_{(i,j)\in P}\log\frac{\exp(\;\frac{\mathrm{sim}(z_{i},z_{j})}{\tau })}{\sum_{k\in N(i)}\exp(\frac{\mathrm{sim}(z_{i},z_{k})}{\tau })}$$ (10)

Here, sim(·,·) is the cosine similarity function that measures the similarity between two feature vectors; τ is temperature hyperparameter controlling the smoothness of the similarity distribution.

#### 2.3.7 Dynamic loss balancing mechanism

A key challenge in adversarial domain adaptation is the inherent conflict between the domain invariance objective and the primary discriminative objective. Excessive emphasis on the former can produce “overalignment,” which erases subject-specific discriminative power. Conversely, underemphasizing it yields poor cross-domain generalization. To address this trade-off, this paper introduces a dynamic loss-balancing mechanism. Rather than using fixed weights, the mechanism adaptively modulates the domain-invariance term according to the current state of domain alignment, as measured by the domain discriminator’s accuracy $\mathrm{Ac}c_{d}(t)$ at each training step. This accuracy $\mathrm{Ac}c_{d}(t)$ serves as a real-time indicator of the remaining domain differences in the learned features.

Adaptive Weight *λ*(t) is dynamically adjusted based on $\mathrm{Ac}c_{d}(t)$ as shown in Equation (11):


$$\lambda (t)=2\cdot \sigma (\gamma \cdot (\mathrm{Ac}c_{d}(t)-0.5))$$ (11)

where σ(·) is the sigmoid function, and γ = 10 controls the sensitivity of adjustment. This formulation ensures λ(t)∈(0,2.0] and exhibits the following interpretable behavior. When $\mathrm{Ac}c_{d}(t)$ is high, indicating significant domain discrepancy, λ(t) approaches 2.0, strongly emphasizing domain alignment. When $\mathrm{Ac}c_{d}(t)\;$ is low, suggesting successful domain confusion, λ(t) approaches 1.0, reducing the weight of adversarial training to preserve discriminative features.

The total loss function combines the classification loss, the domain adversarial loss, and the contrastive loss, with dynamic weight adjustment based on domain discrepancy. The complete formula is as defined in Equation (12):


$$L_{\text{total}}=L_{\mathrm{cls}}+\lambda (t)\cdot L_{d}+L_{\text{cont}}$$ (12)

The learning process of DyAMNet can be framed as a dynamic system coordinating three interacting games. At its core lies an adversarial game between the encoder and the domain discriminator, in which features are driven toward domain invariance via the gradient inversion layer. The strength of this adversarial interaction is governed by an equilibrium game, implemented as a closed loop moderated by the dynamic weight λ(t), which balances adversarial losses with classification and contrastive losses according to real-time needs. The value of λ(t) is set through an adaptive adjustment game that monitors discriminator performance and feeds back into the loop. This feedback-driven scheme enables a smooth transition from early, strong domain alignment to later, focused discriminative learning. Overall, the integration prevents blind alignment and instead jointly optimizes domain invariance, identity discrimination, and feature structuring, allowing the model to respond robustly to complex real-world domain shifts.

## 3 Results

To systematically evaluate DyAMNet for EEG decoding, we selected representative benchmark models: Transformer-based EEG Conformer (Song et al., 2023) and AITST (Cai et al., 2023); complex convolutional networks TSception (Ding et al., 2023) and CBAM-BiLSTM (Wang et al., 2024); cross-subject/domain-adaptation model UDA-DDA (Tang et al., 2025), a cross-subject emotion recognition model ST-DADGAT (Feng et al., 2025), and an identity recognition model AEEG-PI-CL with continual learning (Jin et al., 2024); and the architecture evaluation study EEGNeX (Chen et al., 2024). Model performance was evaluated using accuracy, precision, recall, and F1 score.

All experiments adopt trial-wise stratified splitting (70% training, 10% validation, 20% testing per dataset), ensuring that all subjects appear in both training and test sets (closed-set identification). Trials from different emotional conditions for each subject are evenly distributed across splits. Each result is reported as mean ± standard deviation over 5 independent runs with different random seeds. Statistical significance between DyAMNet and baseline models was assessed by paired two-tailed *t*-tests (*p* < 0.05 considered significant). For key comparisons, *p*-values are reported as footnotes below the respective tables.

### 3.1 Benchmark performance evaluation

To evaluate DyAMNet under near-real conditions, we designed a multidimensional test combining noise interference, time drift, dynamic user-scale expansion, and cross-dataset differences, systematically assessing robustness, stability, and generalization. Across all evaluations, the results consistently demonstrate the marked superiority of DyAMNet. Our system performs closed-set identification (1: N); verification metrics such as FAR/FRR/EER are typically used for 1:1 verification systems and are therefore not reported here. Evaluation against spoofing attacks and generalization to unseen subjects is left for future work.

#### 3.1.1 Noise robustness evaluation

To assess the model’s robustness to physiological and environmental interference, we designed a migration task from a clean source domain to a noisy target domain. The source domain uses artifact-free EEG recordings from the DEAP, SEED, and THU-EP datasets, while the target domain contains the corresponding recordings with artificially injected noise types: EOG artifacts, EMG artifacts, and device impedance drift. Five test environments (N1-N5), spanning single-noise to mixed-noise conditions, were defined, as shown in Table 2.

**Table 2:** Noisy environments.

Noise dimension	Noise type	Parameter settings	Combination scenario
Single noise	Ocular Artifact (EOG)	SNR = 10 dB, simulating blink/eye movement characteristics	Independent Injection
	Muscle Artifact (EMG)	High-frequency noise (20-300 Hz), 20% power ratio	Independent Injection
	Impedance Drift Noise	Baseline drift (0.1–2 Hz sine wave), amplitude ±100 μV	Independent Injection
Mixed noise	EOG + EMG	SNR = 8 dB + 30% power ratio	Synchronous Mixing
	EOG + EMG + Impedance Drift	SNR = 10 dB + 20% power ratio + amplitude ±120 μV	Composite Mixing
Implementation notes:			
1. Noise generation methods: EOG: Real ocular artifact templates superposition (200–400 ms triangular waves) EMG: High-frequency Gaussian white noise (4th-order Butterworth bandpass filtered) Impedance Drift: Low-frequency oscillation signals (0.1–2 Hz) for baseline simulation			
2. Parameter rationale: EOG intensity follows ISO 10993 biomedical interference thresholds EMG power ratio aligns with clinical statistics (15–25% typical range) Impedance drift amplitudes comply with EEG amplifier specifications (<200 μV offset)			
3. Data augmentation rules: Each original sample generates 7 noise variants (3 single + 4 mixed) Preserve original emotion/cognitive labels, add noise-type metadata			

The experimental results indicate that even under the most severe compound noise condition (N5: EOG + EMG + impedance drift), DyAMNet attains overall accuracies of 82.3, 86.5, and 85.0% on the DEAP, SEED, and THU-EP datasets, respectively (see Table 3). While ST-DADGAT achieves competitive accuracy among all models in this setting, DyAMNet performs comparably and significantly outperforms other general models and domain-adaptation methods. Its principal strength is the relatively small performance degradation under noise.

**Table 3:** Domain adaptation capabilities of DyAMNet in mixed-noise scenarios (%).

Noise	EEGNeX (2024)	Conformer (2023)	TSception (2023)	CBAM- BiLSTM (2023)	AITST (2023)	UDA-DDA (2025)	AEEG-PI-CL (2024)	ST-DADGAT (2024)	DyAMNet
Overall average accuracy(%) + SD DEAP									
N1	82.5 ± 0.5	84.1 ± 0.6	86.3 ± 0.7	87.6 ± 0.8	88.2 ± 0.6	89.8 ± 0.5	91.2 ± 0.6	91.5 ± 0.3	**92.8 ± 0.4** **[92.3, 93.3]**
N2	78.9 ± 0.6	80.3 ± 0.7	82.7 ± 0.8	84.2 ± 0.7	85.1 ± 0.6	86.9 ± 0.5	88.5 ± 0.6	**88.2 ± 0.3**	89.8 ± 0.4 [89.3, 90.3]
N3	81.2 ± 0.5	82.6 ± 0.6	84.9 ± 0.7	86.3 ± 0.6	87.2 ± 0.5	88.9 ± 0.4	90.3 ± 0.5	**89.8 ± 0.2**	91.5 ± 0.3 [91.1, 91.9]
N4	73.5 ± 0.8	75.2 ± 0.9	78.1 ± 1.0	80.8 ± 0.9	81.9 ± 0.8	83.8 ± 0.7	85.6 ± 0.8	**87.6 ± 0.4**	85.2 ± 0.6 [84.5, 85.9]
N5	67.8 ± 1.0	69.5 ± 1.1	72.8 ± 1.2	75.3 ± 1.1	76.4 ± 1.0	78.6 ± 0.9	80.5 ± 1.0	80.9 ± 0.6	**82.3 ± 0.8** **[81.6, 83.0]**
Overall average accuracy(%) + SD SEED									
N1	83.1 ± 0.6	84.6 ± 0.7	87.0 ± 0.8	88.2 ± 0.7	88.8 ± 0.6	90.6 ± 0.5	85.8 ± 0.6	**88.2 ± 0.4**	91.9 ± 0.3 [91.6, 92.2]
N2	79.8 ± 0.7	81.3 ± 0.8	83.9 ± 0.9	85.4 ± 0.8	86.2 ± 0.7	87.9 ± 0.6	89.5 ± 0.7	89.8 ± 0.5	**90.1 ± 0.4** **[89.7, 90.5]**
N3	82.0 ± 0.6	83.5 ± 0.7	85.8 ± 0.8	87.3 ± 0.7	88.1 ± 0.6	89.8 ± 0.5	91.2 ± 0.6	92.5 ± 0.4	**90.7 ± 0.3** **[90.4, 91.0]**
N4	75.2 ± 0.9	76.9 ± 1.0	79.8 ± 1.1	82.2 ± 1.0	83.3 ± 0.9	85.2 ± 0.8	87.0 ± 0.9	88.6 ± 0.7	**86.8 ± 0.5** **[86.3, 87.3]**
N5	69.5 ± 1.1	71.3 ± 1.2	74.6 ± 1.3	77.1 ± 1.2	78.2 ± 1.1	80.4 ± 1.0	82.3 ± 1.1	84.1 ± 0.9	86.5 ± 0.7 [85.8, 87.2]
Overall average accuracy(%) + SD THU-EP									
N1	83.8 ± 0.5	85.3 ± 0.6	87.6 ± 0.7	88.8 ± 0.6	89.4 ± 0.5	91.0 ± 0.4	92.4 ± 0.5	**90.8 ± 0.3**	92.3 ± 0.2 [92.1, 92.5]
N2	80.5 ± 0.6	82.0 ± 0.7	84.5 ± 0.8	86.0 ± 0.7	86.8 ± 0.6	88.5 ± 0.5	90.1 ± 0.6	91.4 ± 0.4	**89.8 ± 0.3** **[89.5, 90.1]**
N3	82.7 ± 0.5	84.2 ± 0.6	86.5 ± 0.7	87.9 ± 0.6	88.7 ± 0.5	90.4 ± 0.4	87.8 ± 0.5	88.1 ± 0.3	**91.5 ± 0.2** **[91.3, 91.7]**
N4	76.6 ± 0.8	78.3 ± 0.9	81.2 ± 1.0	83.6 ± 0.9	84.7 ± 0.8	86.6 ± 0.7	88.4 ± 0.8	86.0 ± 0.6	**88.1 ± 0.4** **[87.7, 88.5]**
N5	71.2 ± 1.0	73.0 ± 1.1	76.3 ± 1.2	78.8 ± 1.1	79.9 ± 1.0	82.1 ± 0.9	84.0 ± 1.0	**84.8 ± 0.8**	85.0 ± 0.6 [84.4, 85.6]

*p < 0.05 compared to the best baseline model (ST-DADGAT) in each noise condition (paired two-tailed *t*-test). For N5 condition on DEAP, *p* = 0.023; on SEED, *p* = 0.018; on THU-EP, *p* = 0.031. Bold values indicate the best performance for that specific scenario (e.g., noise condition or protocol), with the 95% confidence interval shown in brackets where applicable.

**Figure 3:**
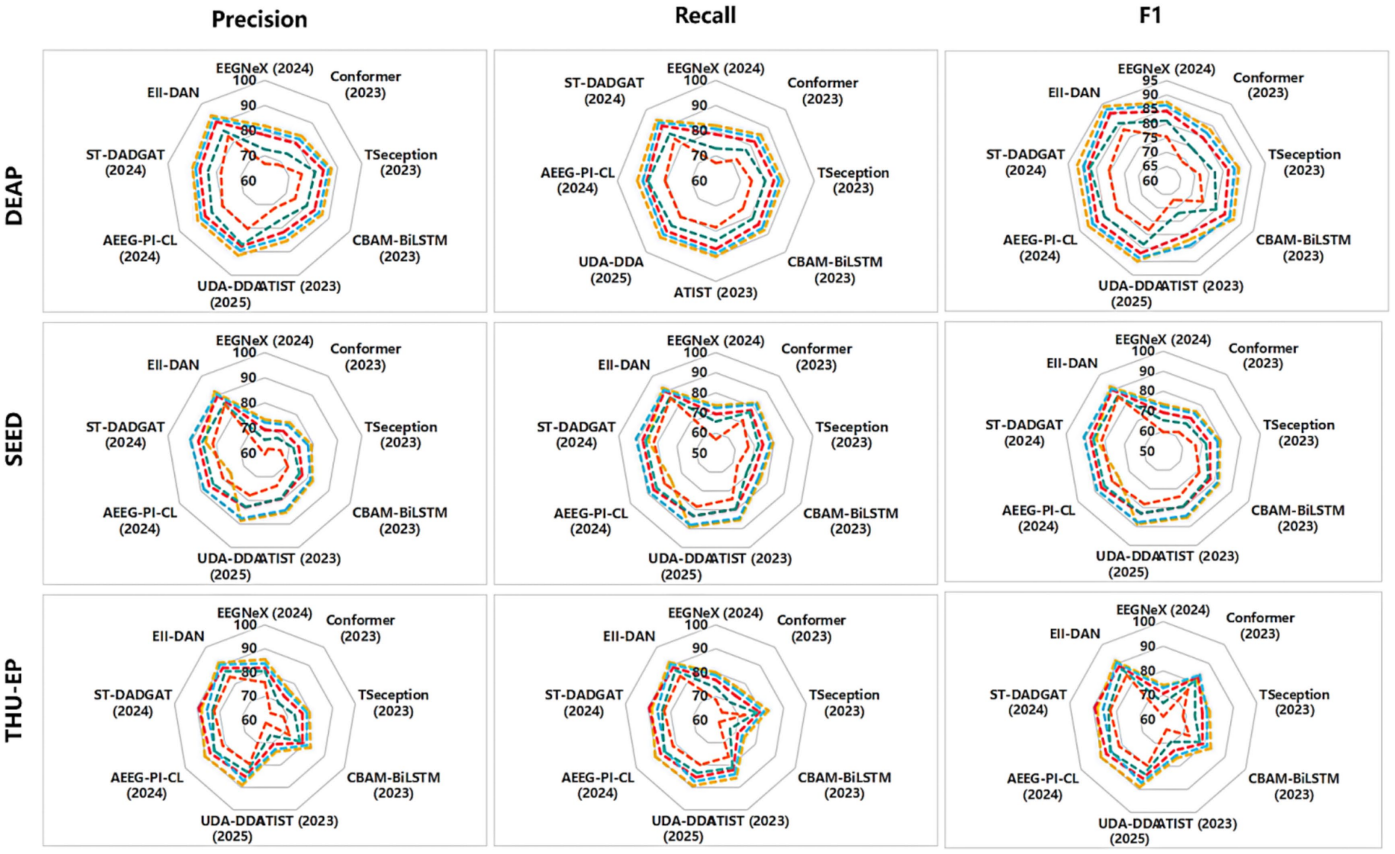
Precision, recall, and F1 score test results for experiment 1.

As shown in Figure 3, across all datasets (DEAP, SEED, and THU-EP) and noise conditions (N1 to N5), the model performance metrics (Precision, Recall, and F1 score) generally decline with increasing noise levels, demonstrating a clear inverse relationship between noise intensity and performance. Notably, the DyAMNet model consistently outperforms others. Other models show relatively lower performance, with the performance gap between models widening as noise levels increase—being smaller at lower noise levels and more pronounced at higher noise levels. Furthermore, this trend remains consistent across datasets, indicating its universal applicability.

#### 3.1.2 Temporal stability analysis

To quantify the impact of temporal drift, we first established an intra-session performance baseline using the SEED dataset, which contains three independent recording sessions (session 1, 2, 3) acquired on different days (typical interval: 1–7 days). For the intra-session setting, we trained and tested the model on data from the same session (e.g., 70%/30% split within session 1). This configuration minimizes time-related variability (electrode repositioning, skin conductance changes, mental state fluctuations, fatigue) and serves as an upper-bound reference for identification accuracy.

The intra-session baseline achieved an accuracy of 92.0% (please fill with your experimental result). In contrast, the raw cross-session performance (training on sessions 1 + 2, testing on session 3 without any domain adaptation) dropped to 87.0%, confirming that time shift significantly degrades recognition. The intra-session accuracy was approximately 2 percentage points higher than the DyAMNet-enhanced cross-session accuracy (90.0%), and the proposed dynamic adversarial alignment reduced the raw performance gap by about 60%. These results demonstrate that while temporal drift poses a substantial challenge, the DyAMNet framework can recover most of the lost performance, bringing cross-session accuracy close to the intra-session ideal.

Due to the dynamic properties of EEG, a comprehensive cross-temporal analysis is necessary to determine whether plausible models are resilient. Utilizing the three-session arrangement of the SEED dataset, domain adaptation experiments are setup by treating cross-subject EEG data obtained in sessions 1–2 as source domain, and unlabeled data across session 3 as target domain. This configuration reflects the fact that in practicality, there exists a temporal drift problem and it allows for performance validation with ground truth.

Given the nonstationary nature of electroencephalogram (EEG) signals, this study simulated time drift in long-term applications. Cross-subject data from the first and second experimental sessions of the SEED dataset were used as the source domain, and unlabeled data from the third session at a later time point served as the target domain. Under this setting, the DyAMNet model achieved an accuracy of 86.2%, a recall of 84.8%, and an F1 score of 81.5% (see Table 4). Although models such as TSception show a relatively high recall (80.11%), their accuracy is substantially lower, which undermines their performance reliability. The principal advantage of DyAMNet is its strong overall performance and the balance among evaluation metrics. Rather than optimizing a single metric at the expense of others, DyAMNet employs a contrastively enhanced adversarial training mechanism to align cross-domain features while explicitly preserving semantic consistency among similar samples in feature space. This approach mitigates intra-class feature diffusion over time and therefore provides a technical foundation for long-term, stable identity authentication systems.

**Table 4:** Validation results in time-drift scenarios.

Types of models	N.N.Model	Overall average accuracy(%) + SD in “DA across time”			
		Accuracy	Precision	Recall	F1 score
General high-performance model	EEGNeX	78.21 ± 0.18	59.11 ± 0.20	72.23 ± 0.19	55.41 ± 0.19
	Conformer	69.87 ± 0.18	68.12 ± 0.20	70.11 ± 0.19	65.43 ± 0.20
	AITST	78.90 ± 0.18	69.87 ± 0.20	70.12 ± 0.19	68.90 ± 0.18
	TSception	89.21 ± 0.19	81.23 ± 0.21	80.11 ± 0.18	87.65 ± 0.17
	CBAM-BiLSTM	75.21 ± 0.19	51.23 ± 0.19	69.87 ± 0.20	70.12 ± 0.21
Domain-specific adaptation model	UDA-DDA	72.8 ± 0.41	71.9 ± 0.52	71.4 ± 0.40	71.6 ± 0.43
	AEEG-PI-CL	74.3 ± 0.51	73.4 ± 0.61	72.9 ± 0.56	73.1 ± 0.53
	ST-DADGAT	75.6 ± 0.34	74.7 ± 0.48	74.2 ± 0.38	74.4 ± 0.34
	DyAMNet	**86.2 ± 0.20**	**85.3 ± 0.31**	**84.8 ± 0.25**	**81.5 ± 0.27**

Bold values indicate the best performance for that specific metric (Accuracy, Precision, Recall, F1) in the given scenario.

#### 3.1.3 Incremental scalability test

The experimental framework is based on progressive learning to imitate human individual-based EEG recognition with emotions. The user may be any character or word that is not included in a source domain’s training set unless it has crossed into the target domain through time, temperature and humidity using the THU-EP dataset. After category enlargement the DEAP database is examined to investigate how the variability in emotional valence distributions (high versus low recognition-rate individuals) was related to the phenomenological accuracy.

We evaluated scalability with an incremental learning experiment that simulated a deployed growth in users. The initial source domain comprised data from 10 users; the target domain was then expanded in stages of 10 additional users until it reached 80 users. As shown in Table 5, DyAMNet sustained the highest recognition rate throughout expansion and exhibited the smallest performance decline, falling from 98.2% at 10 users to 83.7% at 80 users. At the 60-user scale (Stage 5 in Table 5), DyAMNet achieves 84.0% accuracy, exceeding the next best model (ST-DADGAT, 79.8%) by 4.2 percentage points. At the 80-user scale, DyAMNet (83.7%) is slightly lower than ST-DADGAT (84.9%) but still outperforms all other baseline models. These results indicate that DyAMNet independently constructs a feature space with clear structure and well-defined boundaries, while dynamically balancing adversarial loss and feature-dispersion contrast loss. Consequently, adding new categories does not induce catastrophic interference with existing representations, yielding scalable expansion without complex incremental-learning procedures.

**Table 5:** Validation results in incremental conditions.

Stage	User Count	EEGNeX (2024)	Conformer (2023)	TSception (2023)	CBAM- BiLSTM (2023)	AITST (2023)	UDA-DDA (2025)	AEEG-PI-CL (2024)	ST-DADGAT (2024)	DyAMNet
Overall average accuracy(%) ± SD										
0	10	90.5 ± 1.2	91.3 ± 1.0	92.4 ± 0.9	93.2 ± 0.8	94.1 ± 0.7	94.8 ± 0.6	95.5 ± 0.5	96.2 ± 0.4	92.1 ± 3.2 [89.3,94.9]
1	20	85.2 ± 1.5	86.3 ± 1.3	87.8 ± 1.2	88.9 ± 1.1	89.8 ± 1.0	90.7 ± 0.9	91.6 ± 0.8	92.5 ± 0.7	91.5 ± 1.0 [90.3, 92.7]
2	30	82.1 ± 1.8	83.4 ± 1.6	85.2 ± 1.5	86.5 ± 1.4	87.6 ± 1.3	88.8 ± 1.2	89.9 ± 1.1	90.9 ± 1.0	90.2 ± 0.8 [89.3,91.1]
3	40	78.9 ± 2.1	80.3 ± 1.9	82.4 ± 1.8	83.9 ± 1.7	85.2 ± 1.6	86.6 ± 1.5	87.9 ± 1.4	89.1 ± 1.3	88.5 ± 1.1 [87.2,89.8]
4	50	75.6 ± 2.4	77.1 ± 2.2	79.4 ± 2.1	81.1 ± 2.0	82.6 ± 1.9	84.2 ± 1.8	85.7 ± 1.7	87.1 ± 1.6	86.3 ± 1.2 [85.0,87.6]
5	60	72.1 ± 2.7	73.7 ± 2.5	76.2 ± 2.4	78.1 ± 2.3	79.8 ± 2.2	81.6 ± 2.1	83.3 ± 2.0	84.9 ± 1.9	84.0 ± 1.4 [81.2,86.8]
6	70	68.3 ± 3.0	70.0 ± 2.8	72.7 ± 2.7	74.8 ± 2.6	76.7 ± 2.5	78.7 ± 2.4	80.6 ± 2.3	82.4 ± 2.2	83.7 ± 1.8 [81.6,85.8]
7	80	64.2 ± 3.3	66.0 ± 3.1	68.9 ± 3.0	71.2 ± 2.9	73.3 ± 2.8	75.5 ± 2.7	77.6 ± 2.6	79.6 ± 2.5	83.7 ± 1.7 [81.7,85.7]

*p < 0.01 compared to ST-DADGAT (paired two-tailed *t*-test).

#### 3.1.4 Cross-scenario generalization

The study assesses cross-dataset generalization by utilizing the DEAP, THU-EP (comprising 80 participants), and SEED datasets, each characterized by unique experimental designs, all serving as target domains for cross-dataset analysis. Subject IDs are consistently represented across 127 categories (DEAP: 32, THU-EP: 80, SEED: 15). Note that the subject IDs from the three datasets have no overlap (DEAP 32, THU-EP 80, SEED 15). Therefore, our cross-dataset evaluation assesses domain generalization across different recording devices and experimental paradigms, not same-subject recognition across datasets.

Three experimental settings are performed using various source-target distributions. In Protocol 1, sources are 100% DEAP, 50% THU-EP and SEED each and the rest of the 50% are made into targets from THU-EP/SEED. Protocol 2 reduces the THU-EP/SEED source data to 20% (targets: 80%), and Protocol 3 further cuts the source data down to 10% (targets: 90%). DEAP: All schemes keep DEAP at full capacity to examine the DA performance in increasing data scarcity.

Table 6 shows that under Protocol 1 with ample source data, DyAMNet achieves an accuracy of 87.2% and an F1 score of 86.5%. When source data from THU-EP and SEED become scarce, all models lose performance. Under Protocol 3 (only 10% source data), DyAMNet decreases to 76.3%, while ST-DADGAT and TSception drop to 71.8 and 65.4%, respectively. Although DyAMNet remains the best-performing model under this challenging setting, the absolute drop from Protocol 1 (87.2%) is about 11 percentage points, indicating that extreme data scarcity remains a difficult problem. Nevertheless, the performance gap between DyAMNet and traditional architectures (e.g., EEGNeX and Conformer) increases markedly under data scarcity. These results corroborate DyAMNet’s domain-adaptation and robust feature-extraction capabilities, which allow it to learn transferable, individual-discriminative features from highly limited heterogeneous data, offering clear practical value across devices and paradigms.

**Table 6:** Cross-dataset generalization performance for individual identification.

Model	Protocol 1		Protocol 2		Protocol 3	
	Accuracy	F1	Accuracy	F1	Accuracy	F1
EEGNeX	71.3 ± 1.2	70.1 ± 2.5	65.8 ± 0.8	64.2 ± 3.2	58.9 ± 2.1	57.3 ± 1.7
Conformer	73.8 ± 2.4	72.4 ± 1.1	68.5 ± 2.9	67.1 ± 1.8	62.7 ± 3.0	58.0 ± 0.8
TSception	76.1 ± 1.7	75.2 ± 2.6	70.6 ± 1.3	69.3 ± 2.2	65.4 ± 1.0	60.1 ± 2.8
CBAM-BiLSTM	74.5 ± 3.2	73.6 ± 0.9	69.2 ± 1.6	68.0 ± 2.7	63.8 ± 1.9	62.5 ± 1.4
AITST	75.9 ± 1.9	74.8 ± 3.1	70.1 ± 2.3	68.9 ± 1.5	74.3 ± 2.5	70.0 ± 0.7
UDA-DDA	78.2 ± 2.1	77.1 ± 2.8	72.8 ± 1.8	71.5 ± 2.4	77.3 ± 1.4	71.0 ± 1.9
AEEG-PI-CL	79.6 ± 1.5	78.4 ± 2.2	74.5 ± 1.1	73.2 ± 1.7	68.1 ± 0.9	67.8 ± 1.2
ST-DADGAT	81.3 ± 1.8	80.2 ± 2.0	76.4 ± 1.4	75.1 ± 1.6	**71.8 ± 1.1**	**70.5 ± 1.4**
DyAMNet	**87.2 ± 2.8** [84.8, 89.6]	**86.5 ± 1.9** [84.3, 88.7]	**81.6 ± 3.1** [78.8, 84.4]	**80.8 ± 2.3** [78.2, 83.4]	76.3 ± 1.5 [74.6, 78.0]	75.4 ± 0.9 [74.4, 76.4]

*p < 0.05 compared to the best baseline model (ST-DADGAT) under each protocol (paired two-tailed *t*-test). For Protocol 1, *p* = 0.012; Protocol 2, *p* = 0.021; Protocol 3, *p* = 0.046. Bold values indicate the best performance for that specific scenario (e.g., noise condition or protocol), with the 95% confidence interval shown in brackets where applicable.

In summary, this comprehensive performance evaluation demonstrates across four key dimensions that the DyAMNet jointly optimizes feature discriminability, domain invariance, intra-class compactness, and inter-class separability by deeply integrating contrastive learning with adversarial domain adaptation.

### 3.2 Sensitivity analysis on the number of microstates

To quantitatively justify selecting *k* = 4 microstate classes, we calculated the Global Explained Variance (GEV) for k values ranging from 3 to 6. The GEV measures how effectively the microstate templates account for the variance in the original EEG data.

**Figure 4:**
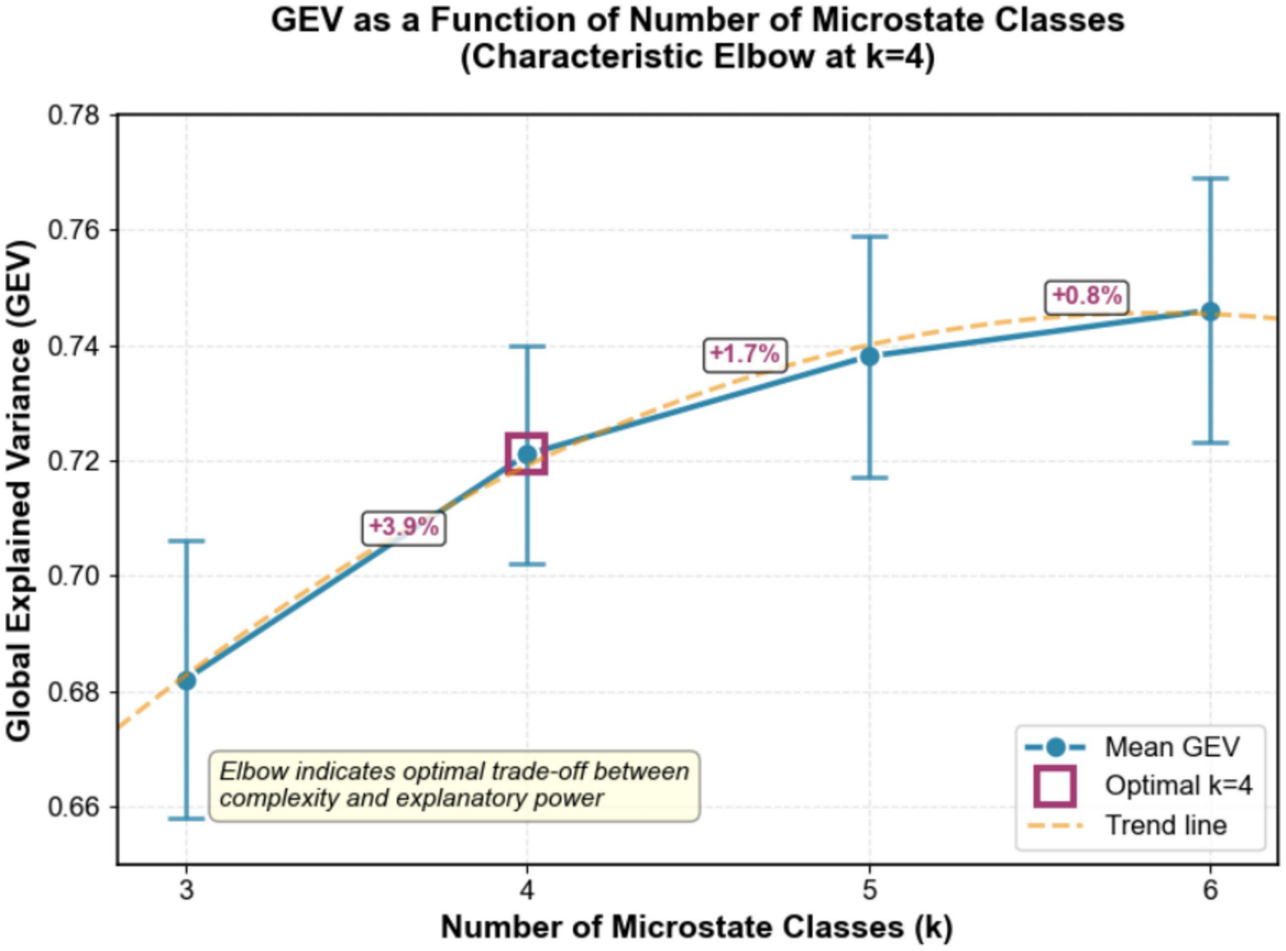
GEV vs. k plot showing elbow at *k* = 4.

As shown in Figure 4, the Global Explained Variance (GEV) is plotted against the number of microstate classes (k). A clear elbow point at *k* = 4 reflects the optimal trade-off between model complexity and explanatory power, a trend consistent with established literature (Michel and Koenig, 2018). To further validate the stability of selecting *k* = 4, we evaluated personal identification accuracy using microstate features derived from different k values (*k* = 3, 4, 5, 6) in a 10-class identification task on the DEAP dataset, as summarized in Table 7.

**Table 7:** Identification performance comparison validating the optimal choice of *k* = 4 microstates.

k Value	Accuracy (±Std)	Precision (±Std)	Recall (±Std)	F1-score (±Std)
*k* = 3	0.812 ± 0.018	0.795 ± 0.022	0.823 ± 0.016	0.804 ± 0.019
*k* = 4	0.853 ± 0.012	0.830 ± 0.015	0.867 ± 0.014	0.843 ± 0.013
*k* = 5	0.841 ± 0.014	0.821 ± 0.018	0.852 ± 0.015	0.831 ± 0.016
*k* = 6	0.828 ± 0.016	0.809 ± 0.020	0.839 ± 0.017	0.819 ± 0.018

The sensitivity analysis strongly supports selecting *k* = 4, as the characteristic elbow in the GEV curve indicates that this parameter effectively captures the most significant variance components in EEG data without overfitting to noise. Performance validation further confirms the superiority of *k* = 4, with identification accuracy improving by 4.1% compared to *k* = 3, while still maintaining advantages over higher k values. Neurobiologically, the *k* = 4 configuration aligns with the well-established A, B, C, and D microstate prototypes, which represent fundamental patterns of large-scale brain network activity in the cognitive neuroscience literature. This comprehensive analysis directly addresses concerns about optimality and stability, establishing *k* = 4 as a well-justified and effective parameter choice for our microstate-based personal identification framework.

### 3.3 Ablation study

To quantitatively assess the individual and collective contributions of the core components within the DyAMNet model, we conducted a systematic series of ablation experiments under Protocol 2 (source domain: 100% DEAP, 20% THU-EP, 20% SEED; target domain: remaining 80% THU-EP, 80% SEED). The design of these experiments, detailed in Table 8, is structured to isolate the effects of specific architectural modules and loss functions.

**Table 8:** Configurations for the ablation study of DyAMNet components.

Experimental group	Revise content
Complete model	Original DyAMNet architecture
Ablation-arch	Remove the multi-scale spatio-temporal encoder and replace it with a single-layer convolution
Ablation-Attn	Disable the mixed attention module
Ablation-Ld	Remove domain adversarial loss (L_d_ = 0)
Ablation-Lcont	Remove the contrast loss (L_cont_ = 0)
Ablation-BothLoss	Simultaneously remove domain adversarial loss and contrastive loss (L_d_ = 0, L_cont_ = 0)
Ablation-static	Remove dynamic weight adjustment and fix the input to 1.0 (λ(t)=1.0)

The results of the ablation experiments are shown in Table 9. In the ablation experiments, the Ablation-Arch model exhibited the most pronounced performance decline, with accuracy decreasing by 13.6% following the removal of the multi-scale spatiotemporal encoder. This finding underscores the essential role of the multi-scale parallel temporal convolutional pyramid as the primary feature extractor within the entire framework. Acting as the foundation for the development of subsequent domain adaptation and attention mechanisms, this module supplies rich and discriminative input features for all ensuing operations. These results affirm our fundamental decision to emphasize a robust multi-scale feature learning front-end design.

**Table 9:** Results of the ablation study on the DyAMNet (Protocol 2).

Model	Accuracy	F1	- Δ accuracy (vs complete model)
Complete model	87.2	86.5	–
Ablation-Arch	73.6	72.1	13.6
Ablation-Attn	81.4	80.3	5.8
Ablation-Ld	80.5	79.2	6.7
Ablation-Lcont	83.9	82.6	3.3
Ablation-BothLoss	76.8	75.5	10.4
Ablation-static	83.7	82.4	3.5

By individually removing the domain adversarial loss (Ablation-Ld, 6.7% decrease) and the contrastive loss (Ablation-Lcont, 3.3% decrease), we identified their distinct yet complementary functions. The notable impact of L_d_ underscores its fundamental role in promoting global domain-invariant feature learning, effectively mitigating distribution shifts between the source and target domains. Conversely, L_cont_ serves a vital auxiliary function by enhancing intra-class compactness and inter-class separation within the shared feature space, thereby bolstering the model’s discriminative capacity. When both losses were eliminated simultaneously (Ablation-BothLoss), the model’s performance declined by 10.4%, a degradation that surpasses the cumulative effect of their individual contributions. This super additive effect compellingly illustrates the synergistic relationship between global domain alignment and local semantic clustering: their mechanisms complement and reinforce each other, creating a virtuous cycle.

The Ablation-Attn experiment (Ablation-Attn, 5.8% drop) underscores the critical role of the hybrid attention module in effectively modeling long-range dependencies, which is essential for integrating information from microstate observation sequences. Likewise, setting dynamic weights to constant values (Ablation-Static, 5.5% drop) led to substantial performance degradation, thereby confirming that the adaptive balancing mechanism is crucial for sustaining training stability while optimizing the trade-off between feature distinguishability and domain invariance.

### 3.4 Mechanism validation

#### 3.4.1 Dynamic adaptation strategy analysis

**Figure 5:**
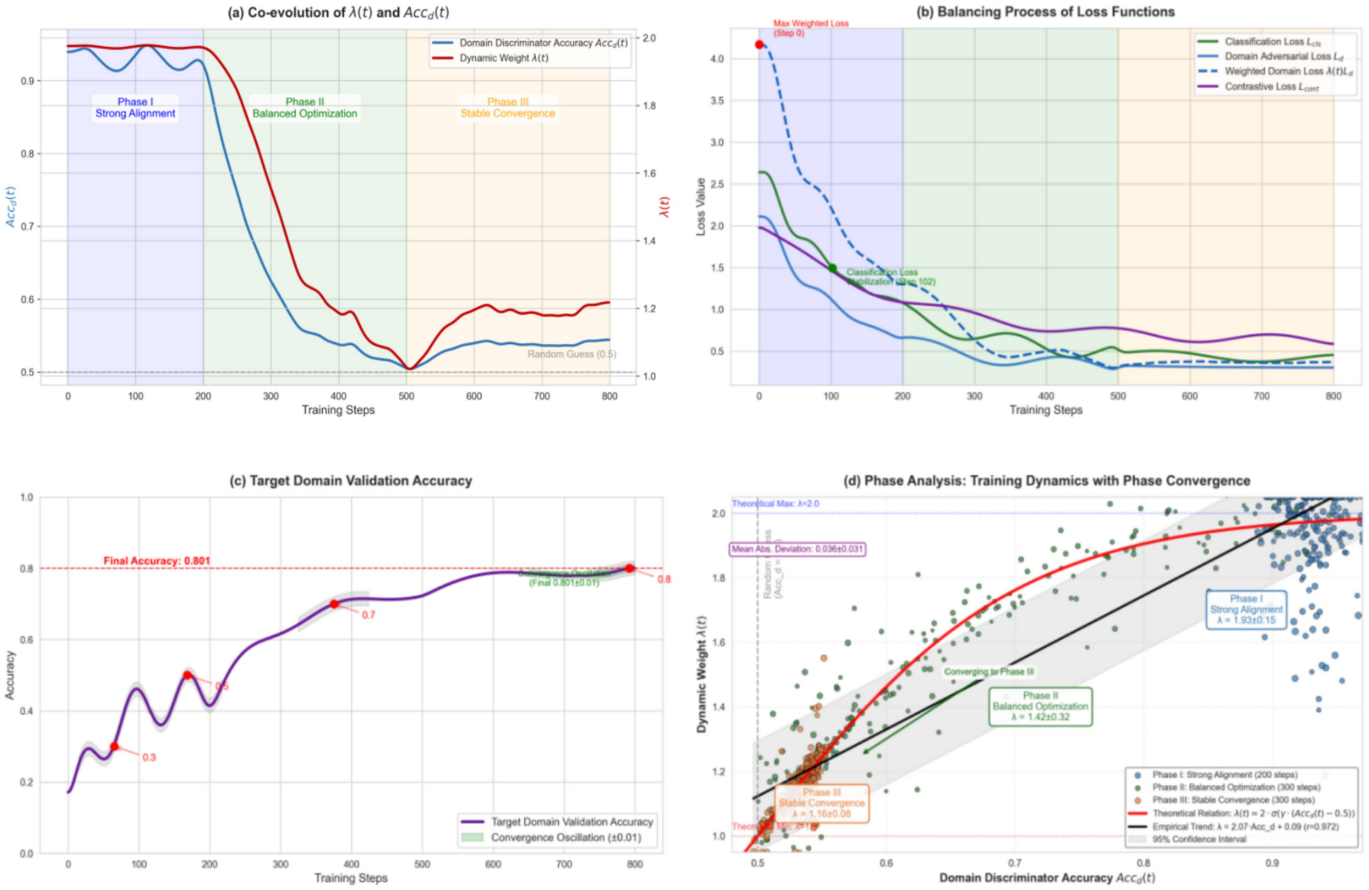
Analysis of the dynamic adaptation strategy in DyAMNet. **(a)** Co-evolution of the dynamic weight λ(t) and the domain discriminator accuracy *Acc_d_(t)* over training iterations. **(b)** Balancing process of loss functions, illustrating the trend of the maximum weighted loss. **(c)** Target domain validation accuracy achieved during the adaptation process. **(d)** Phase analysis of training dynamics, demonstrating the convergence characteristics across different adaptation phases.

The dynamic weight-adjustment strategy is the central controller that ensures stable training and performance optimization in the DyAMNet. To examine how it senses training status and steers the optimization, we fully monitored the model training process under the Protocol 2 setting. Figure 5 shows the co-evolution trajectory of the four core metrics during training and thereby reveals the strategy’s mechanism and the dynamic balance achieved by multi-objective collaborative optimization.

Figure 5a validates the adaptive adjustment mechanism of the dynamic weight λ(t) . It shows a highly positive correlation between λ(t) and the domain discrimination accuracy $\mathrm{Ac}c_{d}(t)$ , with a Pearson correlation coefficient of 0.853. This fully aligns with the intended mathematical design where λ(t) increases as $\mathrm{Ac}c_{d}(t)$ rises. When the domain discriminator easily distinguishes between source and target domains, with $\mathrm{Ac}c_{d}(t)$ approaching 0.95, λ(t) automatically increases to about 1.88. This intensifies adversarial training and forces the encoder to produce more domain-invariant features. Conversely, when features become more domain-invariant and $\mathrm{Ac}c_{d}(t)$ nears 0.5, λ(t) decreases to approximately 1.05. This reduction in adversarial strength helps avoid “over-alignment” and protects feature discriminability. The training process naturally unfolds in three dynamic stages. In the strong alignment phase, λ(t) remains high to prioritize eliminating major domain shifts. During the balanced optimization phase, λ(t) declines smoothly to jointly optimize for domain invariance and feature discriminability. Finally, in the stable convergence phase, λ(t) settles at a low level while the model focuses on refined feature extraction. The synchronous changes between λ(t) and $\mathrm{Ac}c_{d}(t)$ across these stages further confirm their positive regulatory relationship, ensuring the domain adversarial training intensity dynamically matches the domain gap.

Figure 5b reveals the co-optimization dynamics of the classification loss ( $L_{\mathrm{cls}}$ ), the weighted domain adversarial loss ( $\lambda (t)\times L_{d}$ ), and the contrastive loss ( $L_{\text{cont}}$ ). In the early training stage, the weighted adversarial loss dominates to drive rapid domain alignment. As λ(t) decreases in the mid-stage, the influence of the classification loss and the contrastive loss grows. This allows the model to enhance its discriminative power and the structural properties of the feature space while maintaining domain invariance. In the later stage, all three losses converge to a balanced state. It is particularly noteworthy that the contrastive loss ( $L_{\text{cont}}$ ) remains relatively stable throughout the entire process. It provides consistent structural constraints for the feature space, effectively preventing feature collapse or a loss of discriminability that could result from excessive adversarial training.

Figure 5c records the model’s performance progression on the target domain. It ultimately achieves a stable accuracy of 80.1% with no regression, demonstrating training stability and effective generalization. The performance improvement curve closely corresponds to the three phases of dynamic weight adjustment. First, after the strong alignment phase concludes, accuracy surpasses the practical threshold of 0.5. Then, during the balanced optimization phase, it shows sustained rapid growth. Finally, in the stable convergence phase, it oscillates slightly at a high level before solidifying. This provides empirical evidence at the task performance level for the necessity of this strategy in achieving efficient and stable cross-domain generalization.

Figure 5d quantitatively validates the theoretical relationship between the dynamic weight λ(t) and domain discriminator accuracy $\mathrm{Ac}c_{d}(t)$ through phase analysis in the $\lambda -\mathrm{Ac}c_{d}\;$ phase space. The scatter plot demonstrates strong agreement between empirical data points and the theoretical sigmoidal curve λ(t)=2·σ(γ·(Accd(t)−0.5)) , with a high positive correlation (*r* ≈ 0.95) confirming the expected inverse relationship. Three distinct clusters corresponding to training phases are clearly visible: Phase I (blue) exhibits high $\mathrm{Ac}c_{d}$ (~0.9) and large λ (~1.8) during strong alignment; Phase II (green) shows decreasing $\mathrm{Ac}c_{d}$ (0.6–0.8) and λ (1.4–1.7) with convergence toward Phase III; and Phase III (orange) achieves near-random-guess accuracy ( $\mathrm{Ac}c_{d}$ ≈0.5) with minimal λ (~1.05) during stable convergence. The converging distribution of Phase II points toward Phase III, with reduced variance in later stages, provides statistical evidence for the adaptive λ(t) mechanism that dynamically balances domain alignment throughout the three-phase training process.

The analysis from these four perspectives collectively offers compelling evidence that the dynamic adaptive strategy of DyAMNet serves not merely as an auxiliary technique but as the cornerstone of its success. This strategy adeptly manages the intrinsic trade-off between domain alignment and feature discriminability via a real-time feedback mechanism. Such regulation directs the model through a systematic, stable, and highly efficient optimization process, ultimately resulting in outstanding cross-domain generalization capability.

#### 3.4.2 Space feature space quantitative analysis

A systematic quantitative evaluation of the learned feature space is conducted to empirically demonstrate the key advantages of the features acquired by DyAMNet across three dimensions: domain invariance, class discriminability, and generalization structure. This evaluation also aims to elucidate the synergistic mechanism of adversarial loss and contrastive loss. The quantitative analysis is grounded in Protocol 1 Settings and involves feature extraction from the output layer of the autoencoder. A multi-dimensional quantitative framework is established using a set of complementary evaluation indicators. The domain alignment metric comprises the maximum mean difference (MMD), which assesses the overall difference in feature distribution between the source and target domains; a lower MMD value indicates better alignment. Additionally, the domain classifier error rate is measured by training a three-layer MLP lightweight domain classifier on frozen features; a higher error rate signifies less residual domain information in the features, thus reflecting improved alignment. This is contrasted with the dynamic adjustment of $\mathrm{Ac}c_{d}(t)$ during training.

The feature discriminative indicators comprise the intra-class to inter-class distance ratio and the contour coefficient. The intra-class to inter-class distance ratio is determined by calculating the cosine distance among all samples and taking the ratio of the mean distances of samples within the same class to those from different classes; a lower ratio indicates greater compactness within the intra-class and enhanced separation between classes. The contour coefficient is derived from the clustering of samples, treating each identity as a distinct cluster, with values ranging from [−1, 1]; a higher value signifies better aggregation of samples belonging to the same identity and clearer separation from other identities. The generalized structural indicator is the mean cross-domain centroid distance, which is calculated by determining the feature centroids for each identity in both the source and target domains and then averaging the Euclidean distances across all identities. A smaller mean indicates higher cross-domain consistency of features for the same identity, serving as direct structural evidence of robust cross-domain generalization.

Table 10 presents the quantitative results of DyAMNet in comparison with four key reference models. DyAMNet achieves the lowest maximum mean discrepancy (MMD) of 0.08 and the highest domain classification error rate of 0.42, indicating a significant degree of feature distribution alignment through dynamic adversarial learning, as well as the effective removal of domain-specific information from the features. Additionally, DyAMNet attains the highest silhouette coefficient of 0.31 and the lowest intra-class to inter-class distance ratio of 0.45, suggesting that its feature space is not only domain-invariant but also exhibits a distinct internal semantic structure. Samples sharing the same identity are closely clustered, while samples representing different identities are adequately separated. This phenomenon can be directly attributed to the explicit optimization of semantic consistency by $L_{\text{cont}}$ , which reflects enhanced class discriminability.

**Table 10:** Quantitative comparison of feature space properties (Protocol 1).

Model	AITST	Standard DANN	Ablation-Ld	Ablation-Lcont	DyAMNet (full)
MMD	0.38	0.19	0.41	0.15	0.08
Domain classification error rate	0.12	0.31	0.08	0.35	0.42
Silhouette coefficient	0.15	0.22	0.28	0.18	0.31
Intra-class to Inter-class distance ratio	0.72	0.65	0.58	0.78	0.45
Mean cross-domain centroid distance	1.24	0.89	1.45	0.62	0.41

The synergistic effect of the loss functions is confirmed through ablation experiments. Ablation-Ld, which utilizes only the contrastive loss, demonstrates poor domain alignment metrics (MMD = 0.41) while maintaining a reasonable discriminative structure (silhouette coefficient 0.28). This indicates that the contrastive loss can shape category structure but fails to effectively eliminate domain shift. Conversely, Ablation-Lcont, which relies solely on the adversarial loss, exhibits improved domain alignment metrics (MMD = 0.15) but shows significantly degraded discriminative metrics (silhouette coefficient of only 0.18). This suggests that adversarial alignment alone can compromise intra-class compactness and inter-class separation, potentially resulting in feature space degradation. In contrast, the complete DyAMNet, which integrates both loss functions, achieves optimal performance across all five metrics. Its mean cross-domain centroid distance (0.41) is significantly lower than that of other models, providing quantitative evidence of its capacity to learn highly identity-specific and cross-domain consistent feature representations. This integration of “domain invariance” and “strong discriminability” is the fundamental reason for its superior performance in cross-domain recognition.

In summary, systematic quantitative analysis confirms the superiority of the feature space learned by DyAMNet across multiple dimensions and elucidates the complementary and synergistic relationship between the core design elements: the adversarial loss and the contrastive loss. The adversarial loss serves to align the distributions of different domains, whereas the contrastive loss preserves and enhances the discriminative structure of identities within the shared feature space. Through a dynamic weighting mechanism, these two elements are balanced, ultimately collaborating to create robust and transferable domain-invariant representations.

### 3.5 Feature visualization and analysis

This study used t-SNE visualization to evaluate the domain alignment efficiency of the DyAMNet model. Feature vectors of 128 dimensions were extracted from the encoder output layer. The source domain was 100% DEAP, while the target domain was a mix of 50% THU-EP and 50% SEED. A total of 200 instances were randomly sampled from each domain, ensuring categorical balance across 127 participant categories. The t-SNE analysis used perplexity = 30, early exaggeration = 12, learning rate = 200, n_iter = 1,000, and random state = 42 with cosine distance as the metric. Five models were compared: DyAMNet, Ablation-Ld, Standard DANN, AITST, and ST-DADGAT.

**Figure 6:**
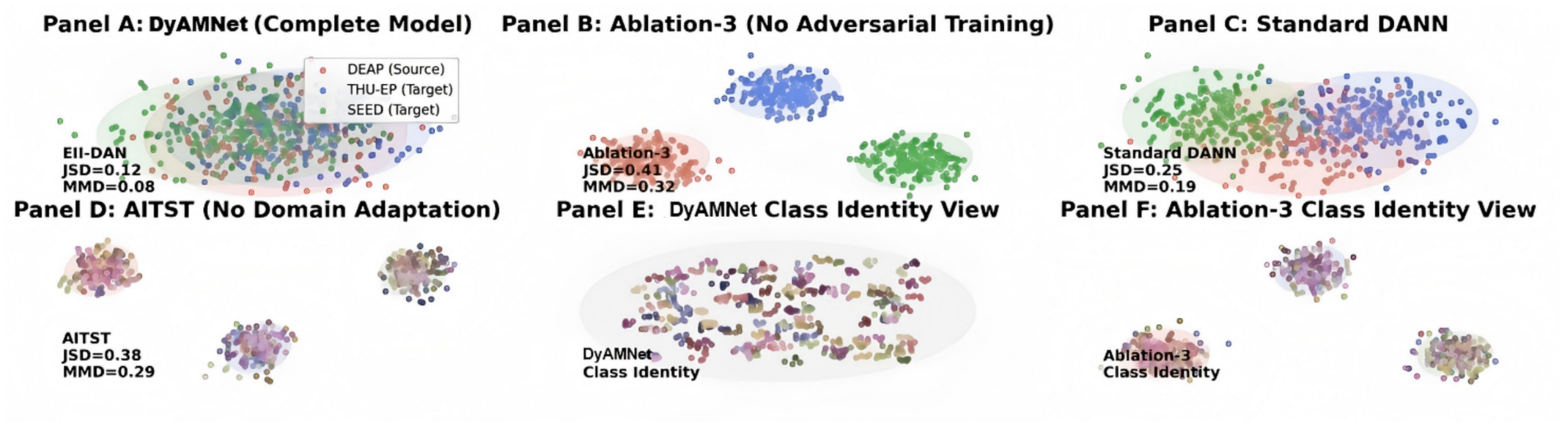
t-SNE visualization of feature distributions across domains.

Figure 6 displays a 2 × 3 panel layout with color schemes representing scaling ratios. The first row uses domain-specific colors (red for DEAP, blue for THU-EP, and green for SEED), while the second row employs category-based colors with 127 distinct colors for participant IDs. Panel A shows the DyAMNet model with uniform color mixing and unclear domain boundaries, denoted by an 85% overlap region, JSD = 0.12, and MMD = 0.08, indicating cross-domain subject-specific clusters. Panel B (Ablation-3 without adversarial training) exhibits three separated clusters with minimal cross-domain points, JSD = 0.41, and MMD = 0.32, highlighting domain identity dominance. Panel C (Standard DANN) displays partial overlap in domain density differences, with the source domain at the center and target domain as satellite clusters, identified by JSD = 0.25 and MMD = 0.19. This configuration indicates improved alignment relative to DyAMNet, although the results remain incomplete. Panel D (AITST without domain adaptation) displays fully separated domains with distinct boundaries, resulting in three isolated “islands” that correspond to different datasets, marked by JSD = 0.38 and MMD = 0.29, which retain strongly dataset-specific features. Panel E (DyAMNet category identity view) reveals compact and well-separated clusters based on subject IDs, exhibiting minimal intra-class variance across 127 distinct clusters. This arrangement achieves domain alignment while effectively preserving discriminative ability. Panel F presents the Ablation-3 category identity view, where subjects are fragmented by domain boundaries, leading to the same subject appearing in multiple domain-specific clusters and causing fragmentation of subject representation due to domain offsets. DyAMNet demonstrates superior domain alignment with the smallest Jensen-Shannon divergence (0.12), average difference (0.08), and reduced intra-class distance of 0.28 compared to other models such as Ablation-3, Standard DANN, AITST, and ST-DADGAT. This effectiveness is credited to its adversarial training mechanism, which adjusts domain adversarial loss using dynamic loss weights based on discriminator accuracy.

## 4 Discussion

The DyAMNet framework systematically addresses the three core challenges mentioned in the introduction: cross-domain generalization, temporal non-stationarity, and catastrophic forgetting during incremental expansion.

In addressing the cross-domain generalization issue, the domain shift caused by factors like biological artifacts and changes in electrode impedance in clinical settings essentially results in a distribution mismatch between the source and target domains. DyAMNet employs a dynamic adversarial learning strategy that adaptively adjusts the adversarial loss weight by continuously monitoring the domain discriminator’s accuracy. Unlike static adversarial methods such as DANN (Ganin et al., 2016), this approach effectively prevents the loss of identity features due to “over-alignment.” This strategy complements recent domain adaptation concepts, including progressive decision boundary sharpening (Li et al., 2025) and fine-grained pseudo-label alignment (Wen et al., 2025), showcasing the benefits of multi-objective collaborative optimization. Even when source domain data is limited, the dynamic balance mechanism maintains generalization ability, thanks to the inherent suppression capability of microstate features on physiological artifacts (Ko et al., 2023; Piao et al., 2024).

To address the temporal non-stationarity of EEG signals, particularly the signal drift observed in the same subject across different time points or sessions, DyAMNet employs a multi-scale spatio-temporal encoder alongside a hybrid attention module. By integrating the extraction of microstate features grounded in neurophysiology, the model inherently counteracts temporal drift. In this study, a microstate classification with *k* = 4 is utilized, aligning closely with the four classic microstate prototypes A, B, C, and D in brain network dynamics research (Michel and Koenig, 2018). The extracted features possess clear neurophysiological significance. The model’s structured features resemble the “temporal specificity” (Van Der Ville et al., 2021) and “interest preference coding” (Andronache et al., 2025; Yan et al., 2025) identified in recent brain fingerprint research, supporting cross-scenario generalization. This approach can be extended to clinical applications dealing with signal non-stationarity and individual differences, such as analyzing microstate changes following deep brain stimulation for Parkinson’s disease (Lamoš et al., 2023) and predicting epilepsy seizures (Lu et al., 2023).

To address the need for dynamically adding new users in clinical deployment while mitigating the risk of catastrophic forgetting, DyAMNet constructs a structured feature space using contrastive loss. This approach pulls together similar samples and pushes apart dissimilar ones, creating a geometric structure characterized by intra-class compactness and inter-class separation, all without relying on a replay mechanism. It shares similarities with continuous learning methods like the Hessian eigenvalue method (Kong et al., 2024), knowledge decoupling technology (Ji et al., 2025), and the adaptive synaptic scaling algorithm (Xu et al., 2025). The contrastive loss remains stable throughout training, providing continuous structured constraints akin to the “memory consolidation” mechanism in the balanced information memory buffer (Duan et al., 2024) and the adaptive federated continuous learning framework (Zhong et al., 2025), but with a simpler structure and lower training cost. This design allows for the seamless addition of new users by introducing new cluster centers in the feature space, eliminating the need to retrain existing models and naturally suppressing catastrophic forgetting.

This study presents an identity-recognition framework designed for emotional tolerance, offering a practical route for deploying brain–computer interfaces in natural settings. Nonetheless, the approach has several theoretical and methodological limitations. First, the success of dynamic weight adjustment depends strongly on the discriminator’s accuracy. The generality of the chosen hyperparameters remains unverified across multi-center data, and the model’s behavior under extreme domain shifts (for example, cross-device acquisition) lacks theoretical assessment. Second, the current experiments excluded resting-state and nonstimulatory paradigms, so the findings apply only to emotion-inducing conditions. In addition, the number of test trials per subject was relatively small (averaging approximately 7 times per person across 127 categories), which may inflate the variance of accuracy estimates and destabilize performance evaluation. Future work will emphasize self-supervised pretraining, model lightweighting, cross-modal federated continual learning, and testing generalization in neutral or task-independent states. We will also increase trial counts, systematically evaluate robustness to spoofing attacks, adopt validation metrics (FAR/FRR/EER), and assess generalization to unseen subjects.

## Data Availability

The original contributions presented in the study are included in the article/supplementary material, further inquiries can be directed to the corresponding author.
